# Disorganized Gut Microbiome Contributed to Liver Cirrhosis Progression: A Meta-Omics-Based Study

**DOI:** 10.3389/fmicb.2018.03166

**Published:** 2018-12-18

**Authors:** Li Shao, Zongxin Ling, Deying Chen, Yufeng Liu, Fengling Yang, Lanjuan Li

**Affiliations:** ^1^State Key Laboratory for Diagnosis and Treatment of Infectious Diseases, Collaborative Innovation Center for Diagnosis and Treatment of Infectious Diseases, The First Affiliated Hospital, School of Medicine, Zhejiang University, Hangzhou, China; ^2^Pharmaceutical Informatics Institute, College of Pharmaceutical Sciences, Zhejiang University, Hangzhou, China

**Keywords:** liver cirrhosis, progression, gut microbiome, metagenomics, metabolomics

## Abstract

Early detection and effective interventions for liver cirrhosis (LC) remain an urgent unmet clinical need. Inspired from intestinal disorders in LC patients, we investigated the associations between gut microbiome and disease progression based on a raw metagenomic dataset of 47 healthy controls, 49 compensated, and 46 decompensated LC patients from our previous study, and a metabolomic dataset of urine samples from the same controls/patients using ultra-performance liquid chromatography/mass spectrophotometry system. It was found that the combination and relative abundance of gut microbiome, the inter-microbiome regulatory networks, and the microbiome-host correlation patterns varied during disease progression. The significant reduction of bacteria involved in fermentation of plant cell wall polysaccharides and resistant starch (such as *Alistipes* sp. *HG5*, *Clostridium thermocellum*) contributed to the reduced supply of energy sources, the disorganized self-feeding and cross-feeding networks and the thriving of some opportunistic pathogens in genus *Veillonella*. The marked decrease of butyrate-producing bacteria and increase of *Ruminococcus gnavus* implicated in degradation of elements from the mucus layer provided an explanation for the impaired intestinal barrier function and systematic inflammation in LC patients. Our results pave the way for further developments in early detection and intervention of LC targeting on gut microbiome.

## Introduction

Cirrhosis is an advanced liver disease with high mortality and morbidity resulting from multiple liver injuries. Determined as the 14th most common cause of death worldwide and fourth most frequent in central Europe, the morbidity and mortality rates due to cirrhosis continue to increase in more developed countries (Tsochatzis et al., 2014). Initially regarded as a single disease entity leading to death, cirrhosis is now increasingly accepted as a dynamic process with the 1 year mortality rate ranging from 1 to 57% for patients at distinct clinical prognostic stages (Tsochatzis et al., 2014). Therefore, development of early interventions to stabilize disease progression and to avoid or delay decompensation of patients is of vital importance. However, chronic liver disease is notoriously asymptomatic in most cases until the occurrence of clinical decompensation. Moreover, few clinical examinations are currently available for diagnosis of cirrhosis, except liver biopsy, which is not applicable to all patients and can lead to various complications in 2–3% patients (Schuppan and Afdhal, 2008). Therefore, clarification of the mechanisms underlying disease progression and identification of appropriate clinical factors that could be utilized as targets for disease monitoring and treatment remain an urgent medical requirement.

Increasing evidence supports the significant association of gut dysbiosis with various kinds of diseases, such as diabetes (Marino et al., 2017), obesity (Bouter et al., 2017), and colorectal cancer (Yu et al., 2017). The liver receives most of its blood supply from the intestine through portal vein and is therefore one of the organs predominantly exposed to potential toxic factors originating from the gut (Llorente and Schnabl, 2015). The gut microbiome has been shown to be involved in the induction and promotion of liver damage in early-stage liver disease (Yan et al., 2011). Enteric dysbiosis, particularly translocation of bacteria and their products through the gut epithelial barrier, plays an important role in the progression and complications of end-stage liver cirrhotic conditions (Lachar and Bajaj, 2016). Previous metagenomic research by our group further confirmed significant dysbiosis of the gut microbiome in LC patients (Qin et al., 2014). Thus, gut microbiome presents a potential target for manipulation to understand and monitor LC progression.

Obligate metabolic interactions exist in natural bacterial communities (Pande and Kost, 2017). Bacteria commonly release metabolites into the external environment, which form an ecological niche benefiting auxotrophic cells that have lost the ability to autonomously produce the corresponding metabolites (Pande and Kost, 2017). The mammalian gut microbiota interacts extensively with the host through metabolic exchange and co-metabolism of substrates, which is implicated in the etiology of many human diseases (Nicholson et al., 2005). These findings indicate critical roles of low molecular weight metabolites of gut microbiome in communicating among bacteria and between bacteria and hosts. Therefore, integrated metabolomic and metagenomic analyses could provide significant advantages in delineating the dynamics and mechanisms of gut microbiome in LC progression. To date, meta-omics-based research has demonstrated considerable benefits in various disease types, including pediatric nonalcoholic fatty liver disease (Del Chierico et al., 2017) and late-onset sepsis in preterm neonates (Stewart et al., 2017).

Several investigations have confirmed dysbiosis of gut microbiome in LC patients (Betrapally et al., 2016; Tilg et al., 2016). However, the contributions of gut microbiome in LC progression, and the feasibility of prevention and early intervention targeting on gut microbiome remain to be established. Based on the huge quantity of metagenomic data already generated for a large number of healthy controls and LC patients and the collection and deposition of urine samples from the same controls/patients in our previous study (Qin et al., 2014), we investigated the dynamics, cross-talk and roles of gut microbiome during clinical progression of LC. The results of this study should pave the way for further researches focusing on monitoring and controlling LC targeting on gut microbiome.

## Materials and Methods

### Study Design

**FIGURE 1:**
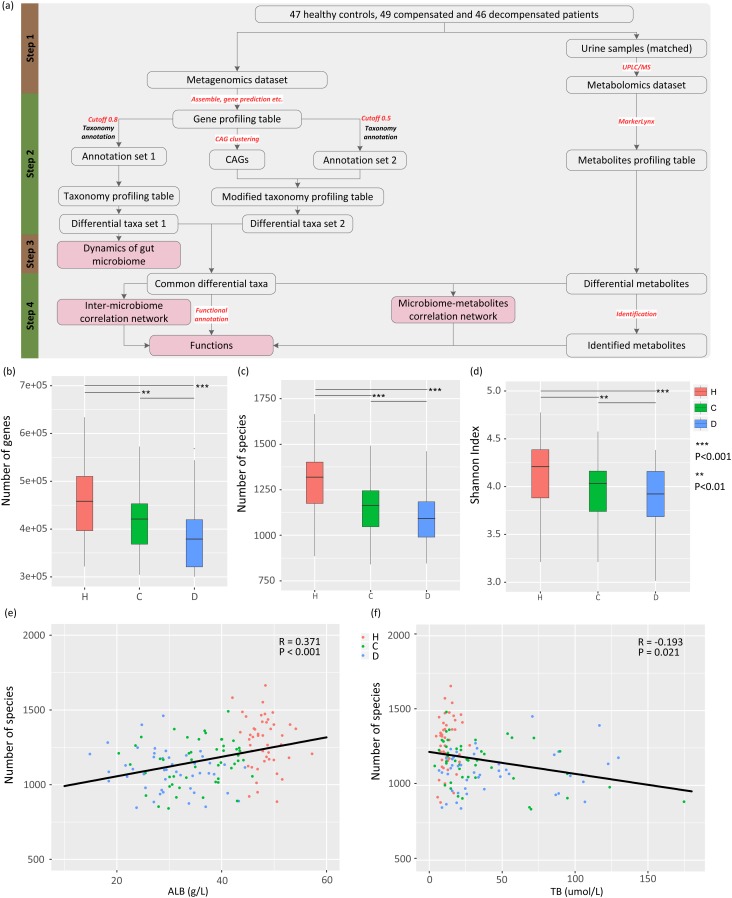
Study design, and associations between gut microbiome and disease progression. **(A)** Schematic diagram of the study design. **(B)** Boxplot of microbial gene richness in healthy controls, compensated and decompensated patients. **(C)** Boxplot of microbial richness in healthy controls, compensated and decompensated patients. **(D)** Boxplot of the Shannon index in healthy controls, compensated and decompensated patients. **(E)** Correlations between microbial richness and albumin. **(F)** Correlations between microbial richness and total bilirubin.

A meta-omics-based study was conducted to investigate the dynamics and roles of gut microbiome in clinical development of LC. The study design was subdivided into four steps (Figure 1A). First, appropriate sets of healthy controls and LC patients with comparable ages were selected from the populations included in our previous study (Qin et al., 2014). Raw metagenomic sequencing data and urine samples from the same healthy controls and/or patients were retrieved, and metabolites in urine samples were analyzed via ultra-performance liquid chromatography/mass spectrum (UPLC/MS) technology. The resulting metabolomic and raw metagenomic datasets generated previously were subsequently processed. Two sets of differential taxa (sets 1 and 2) were obtained using different protocols, as shown Figure 1A. The dynamics of gut microbiome were then profiled from taxa set 1 using a conventional protocol. Finally, differential taxa common in two protocols were selected and used to investigate the inter-microbiome and microbiome–metabolite cross-talk during LC progression. Potential functions associated with the common taxa were also evaluated.

### Description of Samples and the Metagenomic Sequencing Dataset

The raw dataset generated from our previous research (Qin et al., 2014) (ERP005860) was used in this study, which contained paired-end metagenomic sequencing reads for the gut microbiome from fresh stool samples obtained from 123 patients with LC resulting from various kinds of liver injuries and 114 healthy volunteers. In that study, the morning urine samples from the same healthy controls or LC patients were also obtained if available at the same day when stool samples were collected, and were stored at -80°C degrees immediately. In this study, only the metagenomic sequencing reads of LC patients and healthy volunteers with comparable ages were selected. To remove potential confounding factors to the best possible extent, samples with fewer than 11 M sequences were removed. Cirrhotic patients were further classified as “compensated” and “decompensated” according to previously defined principles (Bajaj et al., 2012), resulting in a dataset comprising 47 healthy counterparts (H), 49 compensated (C), and 46 decompensated (D) cirrhotic patients with average ages of 45.34 ± 1.22, 47.69 ± 1.39, and 51.41 ± 1.60 years, respectively. The age was further confirmed not to be a confounding factor in comparisons between healthy controls and compensated patients (HvsC, *P* = 0.21), and between compensated and decompensated patients (CvsD, *P* = 0.08). Detailed information for all participants is provided in a previous report (Qin et al., 2014) and Supplementary Table S1, which also illustrates the availability of urine samples. Our experiments were approved by the Ethics Committee of the First Affiliated Hospital, School of Medicine, Zhejiang University (Zhejiang, China). Informed written consent was obtained from each patient prior to enrollment.

### Construction of a Nonredundant Gene Catalog

Illumina raw paired-end sequencing reads were processed with the MOCAT (Kultima et al., 2012) software package. Briefly, raw sequencing reads were initially filtered using FastX software^1^ with a quality cutoff of 20, and reads shorter than 30 bp discarded. High-quality reads were subjected to human contamination screening. Reads that passed screening were assembled into scaftigs using SOAPdenovo v2.04 (Luo et al., 2012). Genes were predicted from scaftigs longer than 500 bp using MetaGeneMark v3.38 (Besemer and Borodovsky, 1999; Zhu et al., 2010). Redundant genes were removed using CD-HIT (Li and Godzik, 2006) with a cutoff of 90% overlap and 95% identity (no gaps allowed). Finally, cluster representatives shorter than 100 bp were discarded, resulting in 2,332,123 nonredundant genes as the reference gene catalog.

### Quantification of Reference Gene Abundance

High-quality reads that passed human contamination screening were mapped to the reference gene catalog using SOAPaligner v2.21 packed in MOCAT (Kultima et al., 2012) with the following options: –M 4 (find best hits), –l 30 (seed length), –r l (random assignment of multiple hits) and –v 5 (maximum number of mismatches). Mapped reads were subsequently filtered using a cutoff of length 30 bp and 95% identity. The gene length-normalized base counts were calculated using the soap.coverage script^2^. For each sample, 11 M reads (Le Chatelier et al., 2013) were randomly drawn (without replacement) and mapped to the gene catalog to form a downsized depth or abundance matrix.

### Taxonomical Annotation and Abundance Calculation

Catalog genes were assigned taxonomical annotations based on sequence similarity to a database of predicted protein coding genes from 8942 publicly available genomes in the National Center for Biotechnology Information (NCBI, release 196) by MyTaxa (Luo et al., 2014). A likelihood cutoff of 0.8 was applied to determine the taxonomical annotation for the query sequence. The relative abundance of a taxon was calculated as the total relative abundance of genes annotated.

### Construction of Co-abundant Gene Groups and Calculation of Abundance

The canopy-based clustering (Le Chatelier et al., 2013) algorithm with default settings was utilized to generate co-abundant gene groups (CAG) from the reference gene abundance profile across all individuals. MyTaxa (Luo et al., 2014) with a likelihood cutoff of 0.5 was used to determine the taxonomical annotations of genes in each CAG. Only clusters containing more than 50 genes annotated to the same species were retained for further analysis. The abundance of retained CAG was calculated as the mean abundance signal of all genes in each cluster.

### α-Diversity and Gene Count

α-Diversity (within-sample diversity) was calculated from the gene profile of each sample according to the Shannon index as described previously (Qin et al., 2012). In a survey of gene counts, only those with at least one mapped read were considered present (Le Chatelier et al., 2013).

### Gene Functional Classification and Ortholog Group Abundance Profiling

Functional annotation for target genes was achieved with SUPER-FOCUS (Silva et al., 2016). Default settings of parameters were utilized, such as maximum *E*-value 1e^-5^, minimum 60% identity, and minimum alignment length of 15 amino acids. For cases where more than one best hit was found per query sequence, subsystems for all the best hits were retained.

### Urine Metabolomic Analysis and Data Pre-processing

One hundred and seventeen urine samples from the healthy volunteers (*n* = 42) and LC patients (39 compensated and 36 decompensated patients) selected from the above metagenomic analysis procedure were studied in this study. All samples were thawed on ice, vortexed and centrifuged at 14,000 × *g* for 15 min at 4°C. Equal volumes of supernatant (10 μL) from all samples were pooled to obtain quality control (QC) samples. The remaining clear supernatant was placed in UPLC vials for chromatographic separation using a Waters (Milford, MA, United States) ACQUITY UPLC system equipped with an ACQUITY UPLC BEH C18 analytical column. Mass spectrometry was performed on a Waters Q-TOF Premier mass spectrometer in the negative ESI mode. QC samples were injected every six samples throughout the analytical process. The raw UPLC-MS data were processed by MarkerLynx Applications Manager (version 4.1, Waters, Milford, MA, United States), which detected, integrated and normalized the intensities of the peaks to the sum of peaks within the sample, and generated a multivariate dataset based on the retention time, m/z value and signal intensities of the peaks. After partial least squares discriminant analysis (PLS-DA), the differential metabolites were firstly identified by searching MS/MS spectra in the HMDB database^3^. The metabolites that can be preliminarily identified would then be confirmed by corresponding metabolic standards. The metabolite standards methyladenosine, cinnamic acid, phenyllactic acid, decenoylcarnitine, methyluric acid, and alpha-*N*-phenylacetyl-L-glutamine were purchased from Sigma-Aldrich (St. Louis, MO, United States).

### Statistical Analysis

Univariate clinical data are presented as means and standard error of the mean (SEM), and compared between groups by Welch’s *t*-test. Permutational multivariate analysis of variance (PERMANOVA) (Bray–Curtis distance and 9999 permutations for the hypothesis test) and principal co-ordinate analysis (PCoA) based on Bray–Curtis distance were utilized to assess the association between disease progression and gut microbiome. Associations between microbial richness and continuous variables measured clinically were evaluated based on Pearson correlation coefficient. Differences in the relative abundance of taxa and CAGs were identified using LefSe (Segata et al., 2011) with *P* < 0.05 and log-score > 2. Principal component analysis (PCA) and partial least squares discriminant analysis (PLS-DA) of the metabolomics dataset was conducted using SIMCA-P+ 12.0 (Umetrics AB, Sweden) software, and metabolites with variable importance in the projection (VIP) value larger than 1.5 were considered significant. The co-occurrence networks for significant correlations based on permutation analysis (*P* < 0.01) were constructed using the SparCC algorithm (Friedman and Alm, 2012), and visualized by Cytoscape 3.4.0 (Shannon et al., 2003). Differential functions with corrected *P* values less than 0.01 were identified using two-tailed Wilcoxon rank-sum test combined with Benjamini-Hochberg correction (false discovery rate < 0.05). All statistical analyses were conducted using R software (version 3.3.2) unless stated otherwise.

## Results

### Gut Microbial Dysbiosis Is Associated With LC Progression

To delineate the gut microbiome variations associated with LC progression, we retrieved 142 samples from the metagenomic shotgun sequencing data obtained previously (Qin et al., 2014) and acquired taxonomy information using MyTaxa with a likelihood cutoff of 0.8. Microbial gene richness, microbial richness and species diversity decreased significantly (*P* < 0.01) in compensated patients compared to healthy volunteers, and decreased further from compensation to decompensation stages (Figures 1B–D). The number of genes and species, Shannon index for each sample are specified in Supplementary Table S2. PERMANOVA analysis further confirmed significant dysbiosis of gut microbiota in compensated (*P* < 0.001) and decompensated patients (*P* < 0.001) relative to healthy controls, while the variation of gut microbiome during the progression from compensated to decompensated stage was not as marked (*P* = 0.117). A scatter plot on the first three axes of PCoA based on all species (Supplementary Figure S1) further confirmed these findings. Moreover, prominent correlations were observed between microbial richness and clinical indices (Supplementary Table S3), such as albumin (ALB) and total bilirubin (TB) (Figures 1E,F). Our results clearly indicate that gut microbial dysbiosis is related to disease progression.

### Alterations in the Gut Microbiome During LC Progression

**FIGURE 2:**
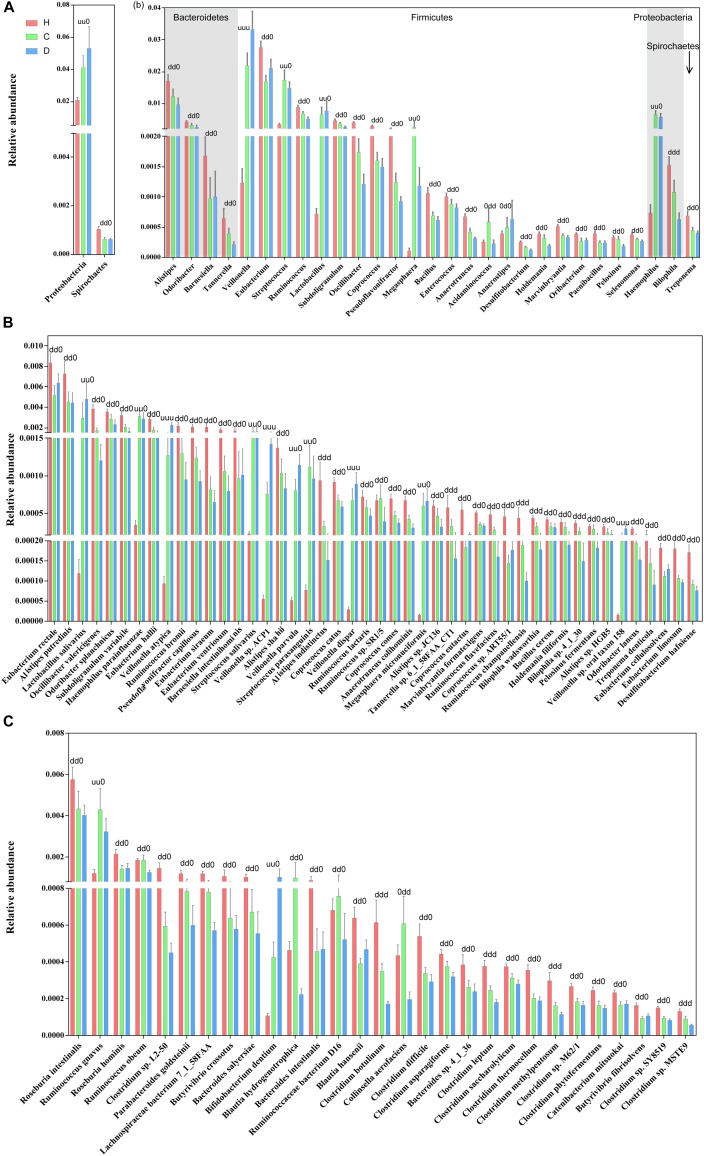
Boxplots of differential gut microbiomes. **(A)** Relative abundance of differential phyla. **(B)** Relative abundance of differential genera. **(C)** Relative abundance of species demonstrating similar alterations with the corresponding genus in both compensated and decompensated patients, compared to healthy controls. **(D)** Relative abundance of species demonstrating different alterations with the corresponding genus in both compensated and decompensated patients, compared to healthy controls. A combination of three characters, “u,” “d,” and “0,” represents the status of compensated and decompensated patients compared to healthy controls and variations during sequential progression from the compensation to decompensation stage. “u,” “d,” and “0” signify significant upregulation and downregulation and no significant variations, respectively.

The relative abundance profiles at the phylum, genus and species levels were further compared. Among the most abundant phyla, *Proteobacteria* and *Spirochaetes* were enriched in patients and controls, respectively, while these alterations in abundance were not so apparent between compensation and decompensation stages (Figure 2A). Twenty-two genera from phyla *Firmicutes*, *Bacteroidetes*, *Proteobacteria*, and *Spirochaetes* (Figure 2B), including *Alistipes*, *Odoribacter*, *Eubacterium*, and *Ruminococcus*, were significantly downregulated, while five (*Veillonella*, *Streptococcus*, *Lactobacillus*, *Megasphaera* and *Haemophilus*) were upregulated in compensated and decompensated patients, compared to healthy controls. It is worth noting that the prominent downregulation of *Tannerella* and *Bilophila* and upregulation of *Veillonella* continued during disease progression from compensation to decompensation stage.

Close inspection revealed that a total of 75 species from phyla *Actinobacteria*, *Bacteroidetes*, *Firmicutes*, *Proteobacteria*, and *Spirochaetes* altered drastically during disease progression. Among these, 46 species (Figure 2C) demonstrated similar alterations with corresponding genus in both compensated and decompensated patients, compared to healthy controls, including 31 with decreased (such as *Eubacterium rectale*, *Alistipes putredinis*, *Alistipes shahii*, and *Coprococcus eutactus*) and 6 with increased (*Haemophilus parainfuenzae*, *Streptococcus salivarius*, *Lactobacillus salivarius*, and *Veillonella parvula*) abundance. Among the remaining 29 species (Figure 2D), 27 including *Roseburia intestinalis*, *Clostridium* sp. *L2-50* and *Bacteroides intestinalis* decreased, while increased levels of *Ruminococcus gnavus* and *Bifidobacterium dentium* were observed in both compensated and decompensated patient groups. During disease progression from compensation to decompensation, *Alistipes indistinctus*, *Bilophila wadsworthia*, *Bilophila* sp. *4_1_30*, *Ruminococcus champanellensis*, *Tannerella* sp. *6_1_58FAA_CT1*, *Clostridium botulinum*, *Clostridium leptum*, *Clostridium methylpentosum* and *Clostridium* sp. *MSTE9* were further downregulated while *Veillonella atypica*, *Veillonella* sp. *ACP1*, *Veillonella dispar*, and *Veillonella* sp. oral taxon 158 were further upregulated. These results support the theory that alterations in the gut microbiome occur in association with liver cirrhosis progression.

### Alterations in Inter-Microbiome Interactions During LC Progression

Inspired by the finding that gut microbiomes potentially act in niche–specific relationships (Nakatsu et al., 2015), we further evaluated inter-microbiome interactions during LC progression. Considering the latent bias involved in library construction, sequencing, and data processing procedures (Goodrich et al., 2014), two protocols were combined to obtain a subset of reliable differential species. Concisely, microbial genes were clustered in to CAG clusters, and those containing more than 50 genes annotated as the same species were retained. The resulting differential species overlapped with those obtained previously were selected for further analysis. Supplementary Figures S2A,B illustrates the phylogenetic tree of the final 30 differential species and the relative abundance using the CAG-based protocol. In total, 21 species, including *E. rectale*, *A. shahii*, and *R. intestinalis*, were downregulated, while 9, including *Veillonella* sp. *ACP1*, *V. atypical*, and *V. dispar*, were upregulated in compensated and decompensated patients, compared to healthy controls.

**FIGURE 3:**
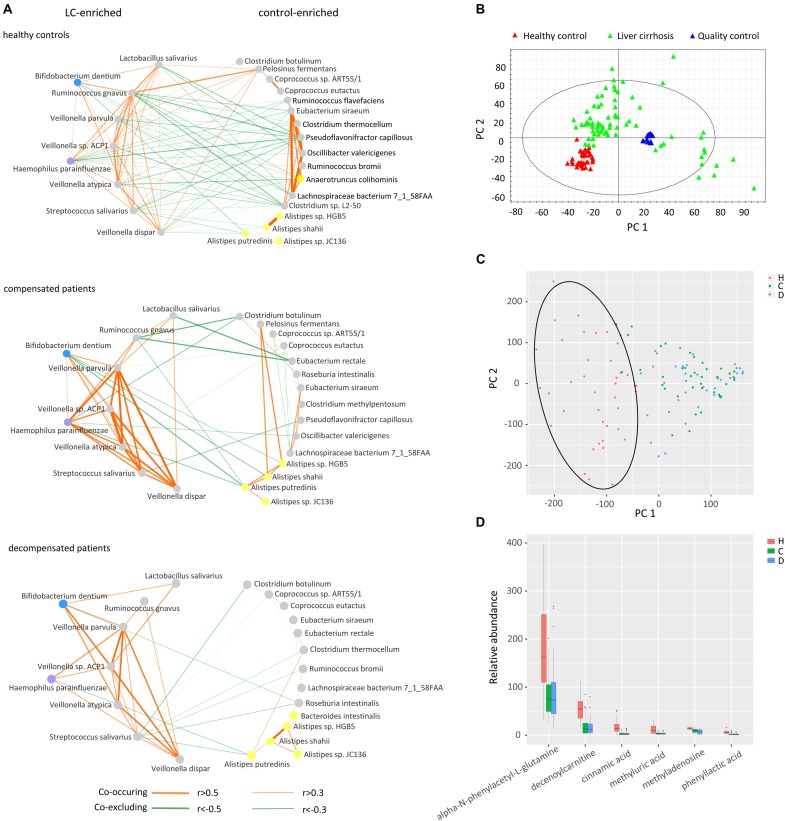
Cross-talk of gut microbiome, score plots of urine metabolites in principal component analysis, and relative abundance of identified metabolites. **(A)** Correlation networks of the gut microbiome in healthy controls, compensated and decompensated patients. Red and green lines represent positive and negative regulatory networks. Nodes filled with gray, yellow, blue, and purple represent species from the phyla *Firmicutes*, *Bacteroidetes*, *Actinobacteria*, and *Proteobacteria*, respectively. **(B)** Score plot of healthy controls, liver cirrhotic patients and quality control samples based on raw metabolomic data without filtering. **(C)** Score plot of healthy controls, compensated and decompensated patients based on 75 differential metabolites. **(D)** Relative abundance of the six metabolites identified in healthy controls, compensated and decompensated patients.

We next inferred all pairwise inter-microbiome correlations in each group of samples. As shown in Figure 3A and Supplementary Table S4, the interactions within the healthy control group were the most extensive and evenly distributed. Broad negative regulatory interactions (53 out of 114 edges) were observed between control-enriched and LC-enriched species. The number of connections was sharply decreased in compensated patients (57 edges) as compared to healthy controls (114 edges). The ratio of negative versus positive edges in compensated patients (17/40) was also significantly lower than that of healthy controls (53/61) (*P* < 0.05). On the other hand, the strength of some interactions, especially the positive associations among LC-enriched *V. parvula*, *Veillonella* sp. *ACP1*, *V. atypica*, *V. dispar*, *S. salivarius*, *B. dentium*, and *H. parainfluenzae*, was drastically enhanced in compensated patients (0.496 ± 0.034) as compared to healthy controls (0.196 ± 0.018) (Supplementary Table S5, *P* < 0.001). During the progression from compensation (57 edges) to decompensation (40 edges), a number of connections were further weakened and/or lost. Most significantly, the negative regulations associated with *R. gnavus* and *L. salivarius* totally disappeared in decompensated patients. These results collectively demonstrate that inter-microbiome interactions altered markedly during disease progression. In conclusion, gut microbial dysbiosis should be evaluated not only for diversity and abundance of microbes but also for inter-microbiome cross-talk.

### Alterations in Microbiome-Metabolite Correlations During LC Progression

The gut microbiome may share metabolites and regulate host metabolism (Pande and Kost, 2017). Accordingly, we further evaluated whether altered inter–microbiome interactions are associated with metabolite exchange by examining the metabolic states of healthy controls and LC patients. As shown in the score plot of PCA based on a total of 10012 peaks (Figure 3B), aggregation of QC samples confirmed the reliability of the metabolomic dataset. After trimming peaks that were absent in 30% samples, 75 metabolites with a VIP value > 1.5 were selected by PLS-DA to be differed among healthy volunteers, compensated and decompensated LC patients. The score plot of PCA based on the 75 metabolites further illustrated that the metabolic state of LC patients was distinct from that of healthy controls while the difference between compensated and decompensated patients was far less significant (Figure 3C). We further identified and confirmed six metabolites by searching MS/MS spectra in the HMDB database^4^ and comparing with those of corresponding standards. The relative abundance of the six metabolites is illustrated in Figure 3D. Detailed information on retention time, molecular weight and VIP value for each of the metabolites is provided in Supplementary Table S6. MS/MS spectra of the six identified metabolites in samples and standards are shown in Supplementary Figure S3.

**FIGURE 4:**
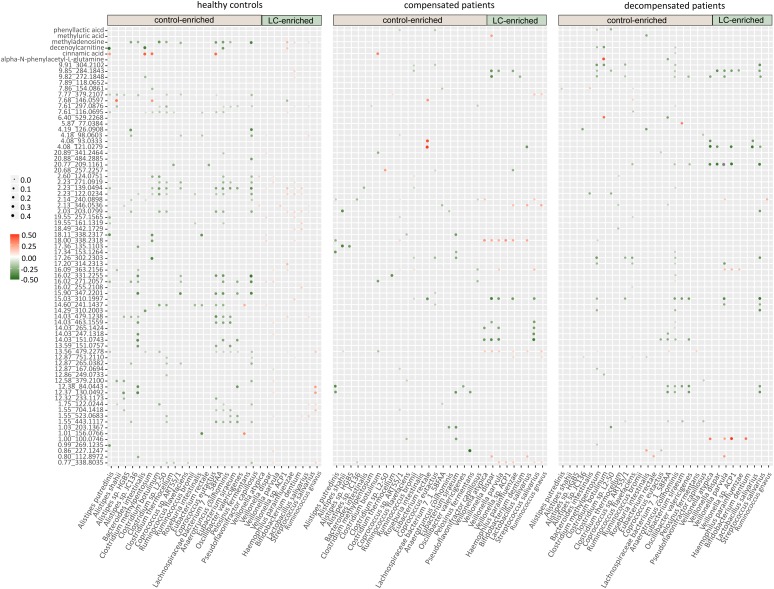
Heatmaps of correlations between the gut microbiome and urine metabolites in healthy controls, compensated and decompensated patients.

We further evaluated the interactions between gut microbiome and urine metabolites in each sample group. Extensive correlations were observed in healthy controls, while the number of associations was markedly lower in both compensated and decompensated patients (Figure 4). Upon separation of the microbiome into control-enriched and LC-enriched components that were down- and up-regulated, respectively, in LC patients relative to healthy controls, several interesting findings were obtained. Most surprisingly, most correlations between control-enriched microbiome and urine metabolites were negative for healthy controls, while nearly all those for the LC-enriched microbiome were positive. During disease progression, different patterns were observed for control- and LC-enriched species. For control-enriched species, the number of connections decreased gradually, especially members of the genus *Alistipes*, which only retained minimal connections in decompensated patients. For LC-enriched species, the pattern changed from most positive correlations in healthy controls to most negative correlations in decompensated patients in addition to a reduced number of connections.

The extensive connections for healthy control-enriched species conformed to high microbial diversity and indicated sharing of metabolites, while the reduced number of connections in compensated and decompensated patients may be associated with downregulation of the corresponding species and thus metabolic capability. The positive correlations observed for LC-enriched species in healthy controls indicates the co-existence of pathogenic species in low abundance in healthy state. The patterns and strength of these connections altered in compensated and decompensated patients probably due to the newly formed niches resulting from microbial dysbiosis. Such phenomenon may either be an underlying reason or result of disordered intestinal microbiota. However, our results clearly support the theory that microbiome–metabolite interactions are altered in association with gut microbial dysbiosis during disease progression.

### Modified Functions of Gut Microbiome During LC Progression

**FIGURE 5:**
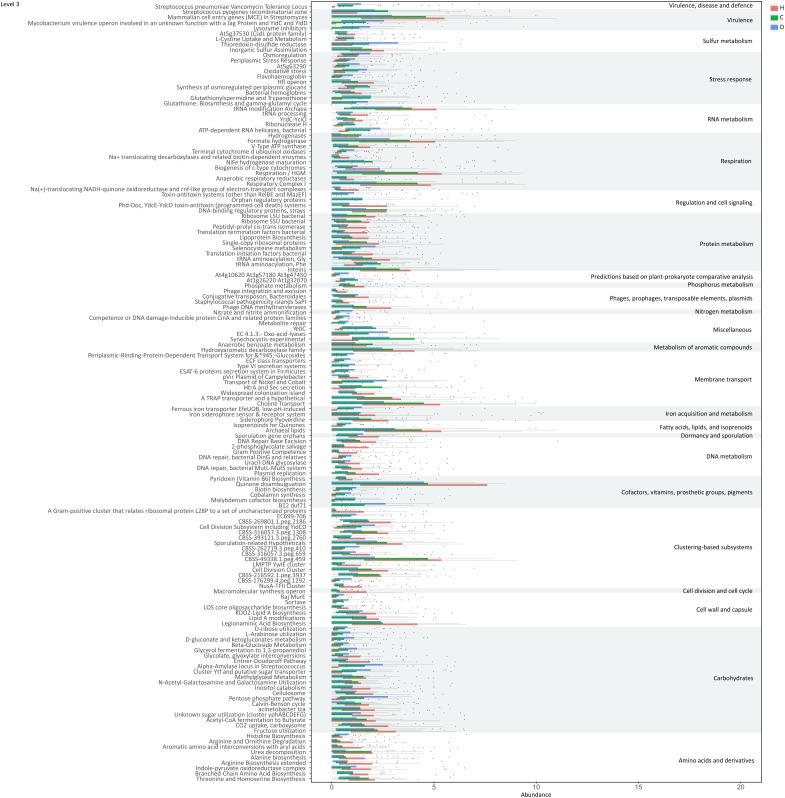
Functions varied during liver cirrhosis progression at level 3.

Functions of genes involved in the 30 differential species were further evaluated based on the SEED database (Silva et al., 2016). Significant variations were observed in multiple functions at various levels during LC progression (Figure 5 and Supplementary Figure S4). The ability of microbiome to degrade plant cell wall polysaccharides was reduced during disease progression, as evident from the decreased function cellulosome complexes, intricate multi-enzyme machines designed by microorganisms for efficient degradation of plant cell wall polysaccharides (Doi and Kosugi, 2004). Meanwhile, tricarboxylate transporter and functions associated with respiration, including respiratory complex I, anaerobic respiratory reductases, ATP synthases and sodium ion-coupled energetics, reduced during disease progression. Functions associated with CO_2_ uptake and fixation and acetyl-CoA fermentation to butyrate were additionally suppressed. With regard to amino acids and derivatives, functions including branched-chain amino acids synthesis, arginine biosynthesis extended, alanine biosynthesis, and urea decomposition decreased, whereas functions such as threonine and homoserine biosynthesis and histidine biosynthesis increased in compensated and decompensated LC patients. Downregulation was observed for functions protein folding, inteins, bacterial translation initiation and translation termination factors, bacterial ribosome small subunit (SSU) and large subunit (LSU), and lipoprotein biosynthesis. Moreover, functions associated with DNA metabolism, such as replication and recombination, were downregulated, while stress responses (including oxidative stress, osmoregulation, periplasmic stress response and others) were upregulated. Detailed information on functions is provided in Supplementary Table S7.

## Discussion

We conducted a meta-omics-based study to evaluate the dynamics and potential roles of gut microbiome in LC progression. It is found that gut microbial gene richness, microbial richness and species diversity decreased, and that patterns of gut microbiome varied during disease progression. Moreover, significant correlations were observed between microbial richness and clinical indices ALB and TB (factors associated with liver function). Thus, we propose that gut microbiome is associated with LC progression.

**FIGURE 6:**
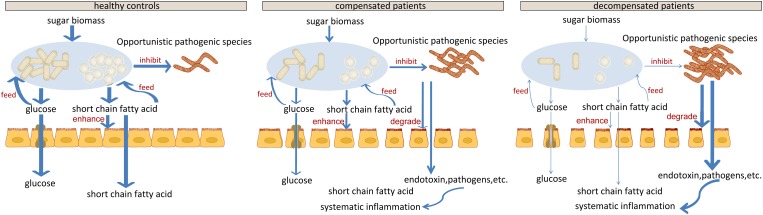
Schematic diagram showing the potential roles of gut microbiota constituents during liver cirrhosis progression.

Our results indicate impaired capability of biomass fermentation in cirrhotic patients, from the gradual decrease of multiple species that can ferment plant cell wall polysaccharides and resistant starch (Xu et al., 2012), and of function cellulosome (Doi and Kosugi, 2004; Figure 5). On the other hand, sugar biomass can be fermented by bacteria with phenylalanine ammonia lyase to phenylalanine as the intermediate and further deaminated to cinnamic acid (Masuo et al., 2016) or decarboxylated to phenylethylamine (Supplementary Figure S5). Phenylethylamine is subsequently metabolized to phenylacetyl-CoA in liver and kidney and subsequently conjugates with glutamine to form phenylacetylglutamine (Aronov et al., 2011). Moreover, glucose, a sugar biomass fermentation intermediate, can be fermented by bacteria with D-phenyllactate reductase to phenylpyruvate and further converted to cinnamic acid by specific species, such as *Clostridium sporogenes* (Masuo et al., 2016). Thus, the sequential decrease in the urine metabolites cinnamic acid, phenylacetylglutamine, and phenyllactic acid (Figure 3D) further confirmed impaired sugar biomass fermentation during LC progression (Figure 6).

Cell-wall polysaccharide and resistant starch generate a variety of metabolites by microbiome, such as glucose and short-chain fatty acids (SCFA). Similar to glucose, SCFAs are also reported to act as energy sources for hosts and play important roles in intestinal homeostasis (Riviere et al., 2015). The gradual decrease in respiration and tricarboxylate transporter-associated functions during LC progression (Figures 5 and Supplementary Figure S4) implied that the impaired sugar biomass fermentation in patients contributed to the reduced supply of the above energy sources, which further contributed to the disorganized self-feeding and cross-feeding networks among microbiota (Figure 3A). The continuous increase in urinary methyladenosine, a modified nucleoside reflecting RNA degradation in the organism (Scorrano et al., 2010), in compensated and decompensated patients might be suggestive of breakdown of microbiota during disease progression. Conversely, some opportunistic pathogens, including *V. atypica*, *V. dispar*, *V. parvula*, and *V.* sp. *ACP1*, thrived during LC progression, which may be explained by reduced suppressive regulations by other dominant bacteria that are downregulated in these patients (Figure 6).

Recent studies suggest that degradation and fermentation of carbohydrates into SCFAs in cross–feeding relationships between microbial groups determine the level of permeability (Chung et al., 2016). Among the SCFAs produced in the human colon, butyrate has drawn the most attention as it is an essential energy source for colon epithelial cells and benefits intestinal barrier function (Kelly et al., 2015). Data from this study showed that butyrate-producing bacteria, such as *E. rectale*, *C. eutacus*, and *Anaerotruncus colihominis*, as well as the function acetyl-CoA fermentation to butyrate (Figure 5) are markedly downregulated in LC patients, compared to healthy controls, while *R. gnavus*, implicated in the degradation of elements from the mucus layer (Graziani et al., 2016), is upregulated. Moreover, it is reported that the gut microbiota resorts to host-secreted mucus glycoproteins as a nutrient source during dietary fiber deficiency, leading to erosion of the colonic mucus barrier (Desai et al., 2016). Thus, reduced biomass fermentation may provide an explanation for impaired intestinal barrier function in LC (Scarpellini et al., 2010; Du Plessis et al., 2013). Under these circumstances, increased infiltration of endotoxins and pathogens from the gut to the peripheral circulation is always accompanied by systematic inflammation (Arroyo et al., 2014). Another report showed that butyrate regulates the proliferation and activation of regulatory T cells in the colon and increases the capacity of regulatory T cells to suppress proliferation of effector CD4+ cells in mice (Nylund et al., 2015). Therefore, reduction of biomass fermentation and the resulting reduced butyrate supply may also be responsible for exacerbation of the infection.

In agreement with the reported increase in bacterial translocation in cirrhotic patients, *B. dentium*, an opportunistic pathogen that mainly inhabits the oral cavity (Xu et al., 2012), and *H. parainfluenzae* residing primarily in the human upper respiratory tract (Young and Hood, 2013) increased during disease progression. Surprisingly, *L. salivarius*, and *S. salivarius* levels also increased during disease progression in the current study. Treatment with dead *L. salivarius* has been shown to decrease intestinal permeability and endotoxin-induced inflammation in diabetic patients (Chung et al., 2016). *S. salivarius* affects immune responses by inhibiting inflammatory pathways activated by pathogens (Kaci et al., 2014), and low molecular-weight metabolites in the culture supernatants of *S. salivarius* are reported to exert *in vitro* anti-inflammatory activity in intestinal epithelial as well as immune cells (Kaci et al., 2011). Therefore, the increase in these two bacteria may indicate upregulated stress response in LC patients, consistent with the upregulation of various functions associated with stress response during disease progression (Figure 5 and Supplementary Figure S4).

## Conclusion

Our data implied a direct link between microbiome changes and LC via metabolites. The metabolic capability of gut microbiome, which played important roles in maintaining the homeostasis of gut microbial system, the normal intestinal barrier function and the immune homeostasis of host, contributed to LC progression. However, due to the limitations of current sequencing and data processing techniques, only partial roles of the gut microbiome have been proposed. With the development of the above techniques, future studies focusing on global microbial system without pre-filtering may provide a more comprehensive picture of roles of gut microbiome, such as the immune response triggered by changes in the microbiota, in LC progression. Detailed resolution of the communication network in gut microbiome may aid in identifying key bacteria, which may be manipulated to slow down and/or reverse the development of LC.

## Availability of Data and Materials

The metagenomic datasets used during the current study are available from the in the European Bioinformatics Institute European Nucleotide Archive under accession number ERP005860 (https://www.ebi.ac.uk/ena/data/view/PRJEB6337).

## Author Contributions

LL, ZL, and LS conceived and designed the experiments. ZL, DC, and FY performed the experiments. LS and YL analyzed the data. LS and ZL wrote the paper and edited the manuscript. The final manuscript was read and approved by all authors.

## Conflict of Interest Statement

The authors declare that the research was conducted in the absence of any commercial or financial relationships that could be construed as a potential conflict of interest.

## Footnotes

1 http://hannonlab.cshl.edu/fastx_toolkit

2 http://soap.genomics.org.cn/down/soap.coverage.tar.gz

3 http://www.hmdb.ca/

4 http://www.hmdb.ca/

## Supplementary Material

FIGURE S1 Scatter plot of healthy controls, compensated and decompensated patients on the first three axes of PCoA analysis. **(A)** Scatter plot of healthy controls and compensated patients. **(B)** Scatter plot of healthy controls and decompensated patients. **(C)** Scatter plot of compensated and decompensated patients. **(D)** Scatter plot of healthy controls, compensated and decompensated patients.

FIGURE S2 Illustration of the final 30 differential species. **(A)** Circular representations of taxonomic and phylogenetic trees of the final 30 differential species. **(B)** Relative abundance of the above 30 species in healthy controls, compensated and decompensated patients based on co-abundant gene clustering analysis.

FIGURE S3 MS/MS spectra of six identified metabolites in samples **(A)** and standards **(B)**.

FIGURE S4 Functions varied during liver cirrhosis progression at level 1 **(A)** and level 2 **(B)**.

FIGURE S5 Schematic illustration of specific pathways in sugar biomass fermentation.

TABLE S1 Detailed clinical information for all patients and healthy volunteers.

TABLE S2 Detailed information on microbial gene richness, gene richness, and Shannon index for each sample.

TABLE S3 Detailed information on the correlation analysis between microbial richness and clinical indices.

TABLE S4 Detailed information on edges of the inter-microbiome correlation networks in each group.

TABLE S5 Detailed information on some edges enhanced in compensated patients as compared to healthy controls.

TABLE S6 Detailed information on retention time, molecular weight and VIP value for 75 differential metabolites.

TABLE S7 Detailed information on functional annotations in various levels.

## References

[B1] Aronov P. A. Luo F. J. Plummer N. S. Quan Z. Holmes S. Hostetter T. H. (2011). Colonic contribution to uremic solutes. *J. Am. Soc. Nephrol.* 22 1769–1776. 10.1681/ASN.2010121220 21784895 PMC3171947

[B2] Arroyo V. Garcia-Martinez R. Salvatella X. (2014). Human serum albumin, systemic inflammation, and cirrhosis. *J. Hepatol.* 61 396–407. 10.1016/j.jhep.2014.04.012 24751830

[B3] Bajaj J. S. O’leary J. G. Reddy K. R. Wong F. Olson J. C. Subramanian R. M. (2012). Second infections independently increase mortality in hospitalized patients with cirrhosis: the North American consortium for the study of end-stage liver disease (NACSELD) experience. *Hepatology* 56 2328–2335. 10.1002/hep.25947 22806618 PMC3492528

[B4] Besemer J. Borodovsky M. (1999). Heuristic approach to deriving models for gene finding. *Nucleic Acids Res.* 27 3911–3920. 10.1093/nar/27.19.3911 10481031 PMC148655

[B5] Betrapally N. S. Gillevet P. M. Bajaj J. S. (2016). Changes in the intestinal microbiome and alcoholic and nonalcoholic liver diseases: causes or effects? *Gastroenterology* 150 1745–1755. 10.1053/j.gastro.2016.02.073. 26948887 PMC5026236

[B6] Bouter K. E. Van Raalte D. H. Groen A. K. Nieuwdorp M. (2017). Role of the gut microbiome in the pathogenesis of obesity and obesity-related metabolic dysfunction. *Gastroenterology* 152 1671–1678. 10.1053/j.gastro.2016.12.048 28192102

[B7] Chung P. H. Wu Y. Y. Chen P. H. Fung C. P. Hsu C. M. Chen L. W. (2016). *Lactobacillus salivarius* reverse diabetes-induced intestinal defense impairment in mice through non-defensin protein. *J. Nutr. Biochem.* 35 48–57. 10.1016/j.jnutbio.2016.05.013 27376728

[B8] Del Chierico F. Nobili V. Vernocchi P. Russo A. Stefanis C. Gnani D. (2017). Gut microbiota profiling of pediatric nonalcoholic fatty liver disease and obese patients unveiled by an integrated meta-omics-based approach. *Hepatology* 65 451–464. 10.1002/hep.28572 27028797

[B9] Desai M. S. Seekatz A. M. Koropatkin N. M. Kamada N. Hickey C. A. Wolter M. (2016). A dietary fiber-deprived gut microbiota degrades the colonic mucus barrier and enhances pathogen susceptibility. *Cell* 167 1339–1353. 10.1016/j.cell.2016.10.043. 27863247 PMC5131798

[B10] Doi R. H. Kosugi A. (2004). Cellulosomes: plant-cell-wall-degrading enzyme complexes. *Nat. Rev. Microbiol.* 2 541–551. 10.1038/nrmicro925 15197390

[B11] Du Plessis J. Vanheel H. Janssen C. E. Roos L. Slavik T. Stivaktas P. I. (2013). Activated intestinal macrophages in patients with cirrhosis release NO and IL-6 that may disrupt intestinal barrier function. *J. Hepatol.* 58 1125–1132. 10.1016/j.jhep.2013.01.038 23402745

[B12] Friedman J. Alm E. J. (2012). Inferring correlation networks from genomic survey data. *PLoS Comput. Biol.* 8:e1002687. 10.1371/journal.pcbi.1002687 23028285 PMC3447976

[B13] Goodrich J. K. Di Rienzi S. C. Poole A. C. Koren O. Walters W. A. Caporaso J. G. (2014). Conducting a microbiome study. *Cell* 158 250–262. 10.1016/j.cell.2014.06.037 25036628 PMC5074386

[B14] Graziani F. Pujol A. Nicoletti C. Dou S. Maresca M. Giardina T. (2016). Ruminococcus gnavus E1 modulates mucin expression and intestinal glycosylation. *J. Appl. Microbiol.* 120 1403–1417. 10.1111/jam.13095 26868655

[B15] Kaci G. Goudercourt D. Dennin V. Pot B. Dore J. Ehrlich S. D. (2014). Anti-inflammatory properties of *Streptococcus salivarius*, a commensal bacterium of the oral cavity and digestive tract. *Appl. Environ. Microbiol.* 80 928–934. 10.1128/AEM.03133-13 24271166 PMC3911234

[B16] Kaci G. Lakhdari O. Dore J. Ehrlich S. D. Renault P. Blottiere H. M. (2011). Inhibition of the NF-kappaB pathway in human intestinal epithelial cells by commensal *Streptococcus salivarius*. *Appl. Environ. Microbiol.* 77 4681–4684. 10.1128/AEM.03021-10 21602373 PMC3127691

[B17] Kelly C. J. Zheng L. Campbell E. L. Saeedi B. Scholz C. C. Bayless A. J. (2015). Crosstalk between microbiota-derived short-chain fatty acids and intestinal epithelial hif augments tissue barrier function. *Cell Host Microbe* 17 662–671. 10.1016/j.chom.2015.03.005 25865369 PMC4433427

[B18] Kultima J. R. Sunagawa S. Li J. Chen W. Chen H. Mende D. R. (2012). MOCAT: a metagenomics assembly and gene prediction toolkit. *PLoS One* 7:e47656. 10.1371/journal.pone.0047656 23082188 PMC3474746

[B19] Lachar J. Bajaj J. S. (2016). Changes in the microbiome in cirrhosis and relationship to complications: hepatic encephalopathy, spontaneous bacterial peritonitis, and sepsis. *Semin. Liver Dis.* 36 327–330. 10.1055/s-0036-1593881 27997972

[B20] Le Chatelier E. Nielsen T. Qin J. Prifti E. Hildebrand F. Falony G. (2013). Richness of human gut microbiome correlates with metabolic markers. *Nature* 500 541–546. 10.1038/nature12506 23985870

[B21] Li W. Godzik A. (2006). Cd-hit: a fast program for clustering and comparing large sets of protein or nucleotide sequences. *Bioinformatics* 22 1658–1659. 10.1093/bioinformatics/btl158 16731699

[B22] Llorente C. Schnabl B. (2015). The gut microbiota and liver disease. *Cell Mol. Gastroenterol. Hepatol.* 1 275–284. 10.1016/j.jcmgh.2015.04.003 26090511 PMC4467911

[B23] Luo C. Rodriguez R. L. Konstantinidis K. T. (2014). MyTaxa: an advanced taxonomic classifier for genomic and metagenomic sequences. *Nucleic Acids Res.* 42 e73. 10.1093/nar/gku169 24589583 PMC4005636

[B24] Luo R. Liu B. Xie Y. Li Z. Huang W. Yuan J. (2012). SOAPdenovo2: an empirically improved memory-efficient short-read de novo assembler. *Gigascience* 1:18. 10.1186/2047-217X-1-18 23587118 PMC3626529

[B25] Marino E. Richards J. L. Mcleod K. H. Stanley D. Yap Y. A. Knight J. (2017). Gut microbial metabolites limit the frequency of autoimmune T cells and protect against type 1 diabetes. *Nat. Immunol.* 18 552–562. 10.1038/ni.3713 28346408

[B26] Masuo S. Kobayashi Y. Oinuma K. Takaya N. (2016). Alternative fermentation pathway of cinnamic acid production via phenyllactic acid. *Appl. Microbiol. Biotechnol.* 100 8701–8709. 10.1007/s00253-016-7623-4 27225472

[B27] Nakatsu G. Li X. Zhou H. Sheng J. Wong S. H. Wu W. K. (2015). Gut mucosal microbiome across stages of colorectal carcinogenesis. *Nat. Commun.* 6:8727. 10.1038/ncomms9727 26515465 PMC4640069

[B28] Nicholson J. K. Holmes E. Wilson I. D. (2005). Gut microorganisms, mammalian metabolism and personalized health care. *Nat. Rev. Microbiol.* 3 431–438. 10.1038/nrmicro1152 15821725

[B29] Nylund L. Nermes M. Isolauri E. Salminen S. De Vos W. M. Satokari R. (2015). Severity of atopic disease inversely correlates with intestinal microbiota diversity and butyrate-producing bacteria. *Allergy* 70 241–244. 10.1111/all.12549 25413686

[B30] Pande S. Kost C. (2017). Bacterial unculturability and the formation of intercellular metabolic networks. *Trends Microbiol.* 25 349–361. 10.1016/j.tim.2017.02.015 28389039

[B31] Qin J. Li Y. Cai Z. Li S. Zhu J. Zhang F. (2012). A metagenome-wide association study of gut microbiota in type 2 diabetes. *Nature* 490 55–60. 10.1038/nature11450 23023125

[B32] Qin N. Yang F. Li A. Prifti E. Chen Y. Shao L. (2014). Alterations of the human gut microbiome in liver cirrhosis. *Nature* 513 59–64. 10.1038/nature13568 25079328

[B33] Riviere A. Gagnon M. Weckx S. Roy D. De Vuyst L. (2015). Mutual Cross-feeding interactions between *Bifidobacterium longum* subsp. longum NCC2705 and Eubacterium rectale ATCC 33656 Explain the Bifidogenic and Butyrogenic Effects of Arabinoxylan Oligosaccharides. *Appl. Environ. Microbiol.* 81 7767–7781. 10.1128/AEM.02089-15 26319874 PMC4616955

[B34] Scarpellini E. Valenza V. Gabrielli M. Lauritano E. C. Perotti G. Merra G. (2010). Intestinal permeability in cirrhotic patients with and without spontaneous bacterial peritonitis: is the ring closed? *Am. J. Gastroenterol.* 105 323–327. 10.1038/ajg.2009.558 19844200

[B35] Schuppan D. Afdhal N. H. (2008). Liver cirrhosis. *Lancet* 371 838–851. 10.1016/S0140-6736(08)60383-9 18328931 PMC2271178

[B36] Scorrano S. Longo L. Vasapollo G. (2010). Molecularly imprinted polymers for solid-phase extraction of 1-methyladenosine from human urine. *Anal. Chim. Acta* 659 167–171. 10.1016/j.aca.2009.11.046 20103120

[B37] Segata N. Izard J. Waldron L. Gevers D. Miropolsky L. Garrett W. S. (2011). Metagenomic biomarker discovery and explanation. *Genome Biol.* 12:R60. 10.1186/gb-2011-12-6-r60 21702898 PMC3218848

[B38] Shannon P. Markiel A. Ozier O. Baliga N. S. Wang J. T. Ramage D. (2003). Cytoscape: a software environment for integrated models of biomolecular interaction networks. *Genome Res.* 13 2498–2504. 10.1101/gr.1239303 14597658 PMC403769

[B39] Silva G. G. Green K. T. Dutilh B. E. Edwards R. A. (2016). SUPER-FOCUS: a tool for agile functional analysis of shotgun metagenomic data. *Bioinformatics* 32 354–361. 10.1093/bioinformatics/btv584 26454280 PMC4734042

[B40] Stewart C. J. Embleton N. D. Marrs E. C. L. Smith D. P. Fofanova T. Nelson A. (2017). Longitudinal development of the gut microbiome and metabolome in preterm neonates with late onset sepsis and healthy controls. *Microbiome* 5:75. 10.1186/s40168-017-0295-1 28701177 PMC5508794

[B41] Tilg H. Cani P. D. Mayer E. A. (2016). Gut microbiome and liver diseases. *Gut* 65 2035–2044. 10.1136/gutjnl-2016-312729 27802157

[B42] Tsochatzis E. A. Bosch J. Burroughs A. K. (2014). Liver cirrhosis. *Lancet* 383 1749–1761. 10.1016/S0140-6736(14)60121-5 24480518

[B43] Xu M. Wang B. Fu Y. Chen Y. Yang F. Lu H. (2012). Changes of fecal Bifidobacterium species in adult patients with hepatitis B virus-induced chronic liver disease. *Microb. Ecol.* 63 304–313. 10.1007/s00248-011-9925-5 21814872

[B44] Yan A. W. Fouts D. E. Brandl J. Starkel P. Torralba M. Schott E. (2011). Enteric dysbiosis associated with a mouse model of alcoholic liver disease. *Hepatology* 53 96–105. 10.1002/hep.24018 21254165 PMC3059122

[B45] Young R. E. Hood D. W. (2013). Haemophilus parainfluenzae has a limited core lipopolysaccharide repertoire with no phase variation. *Glycoconj. J.* 30 561–576. 10.1007/s10719-012-9455-5 23093380

[B46] Yu J. Feng Q. Wong S. H. Zhang D. Liang Q. Y. Qin Y. (2017). Metagenomic analysis of faecal microbiome as a tool towards targeted non-invasive biomarkers for colorectal cancer. *Gut* 66 70–78. 10.1136/gutjnl-2015-309800 26408641

[B47] Zhu W. Lomsadze A. Borodovsky M. (2010). Ab initio gene identification in metagenomic sequences. *Nucleic Acids Res.* 38 e132. 10.1093/nar/gkq275 20403810 PMC2896542

