# Supplementary figures and images for: Disorganized Gut Microbiome Contributed to Liver Cirrhosis Progression: A Meta-Omics-Based Study
Source: Front Microbiol. 2018 Dec 18;9:3166. doi: 10.3389/fmicb.2018.03166 (PMC6315199; doi:10.3389/fmicb.2018.03166)

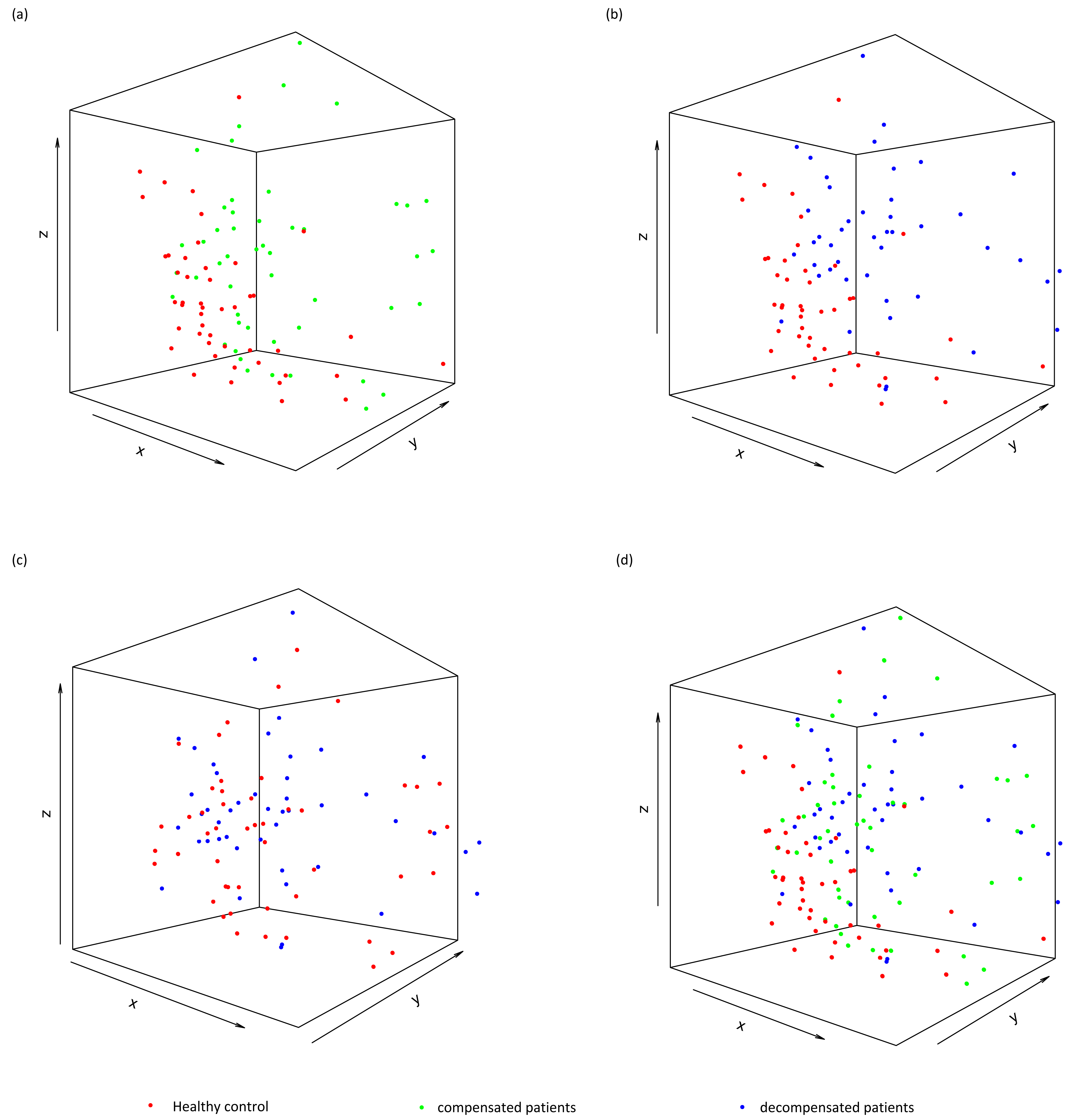

Supplement: FIGURE S1 — Scatter plot of healthy controls, compensated and decompensated patients on the first three axes of PCoA analysis. (A) Scatter plot of healthy controls and compensated patients. (B) Scatter plot of healthy controls and decompensated patients. (C) Scatter plot of compensated and decompensated patients. (D) Scatter plot of healthy controls, compensated and decompensated patients. [file Image_1.TIF]

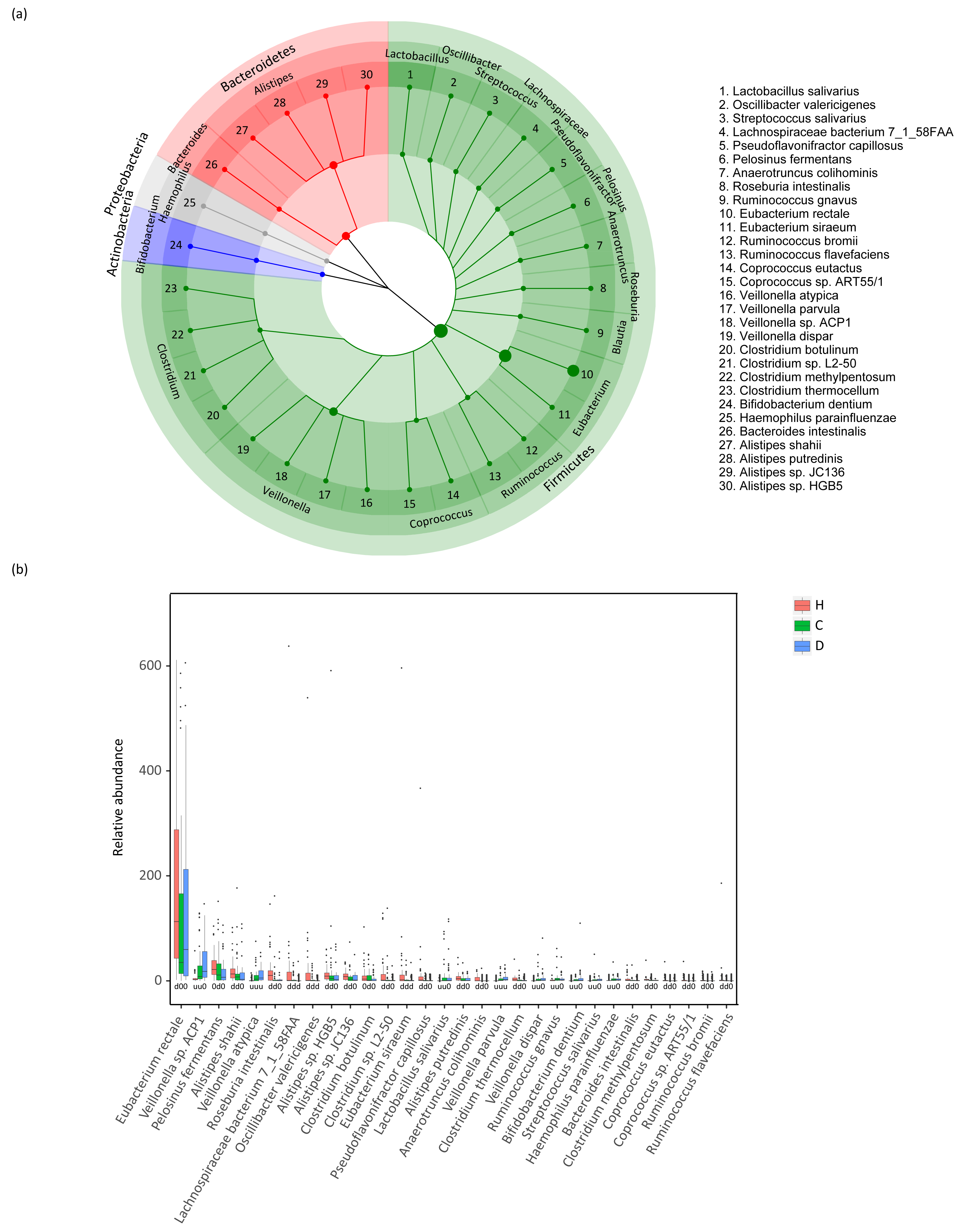

Supplement: FIGURE S2 — Illustration of the final 30 differential species. (A) Circular representations of taxonomic and phylogenetic trees of the final 30 differential species. (B) Relative abundance of the above 30 species in healthy controls, compensated and decompensated patients based on co-abundant gene clustering analysis. [file Image_2.TIF]

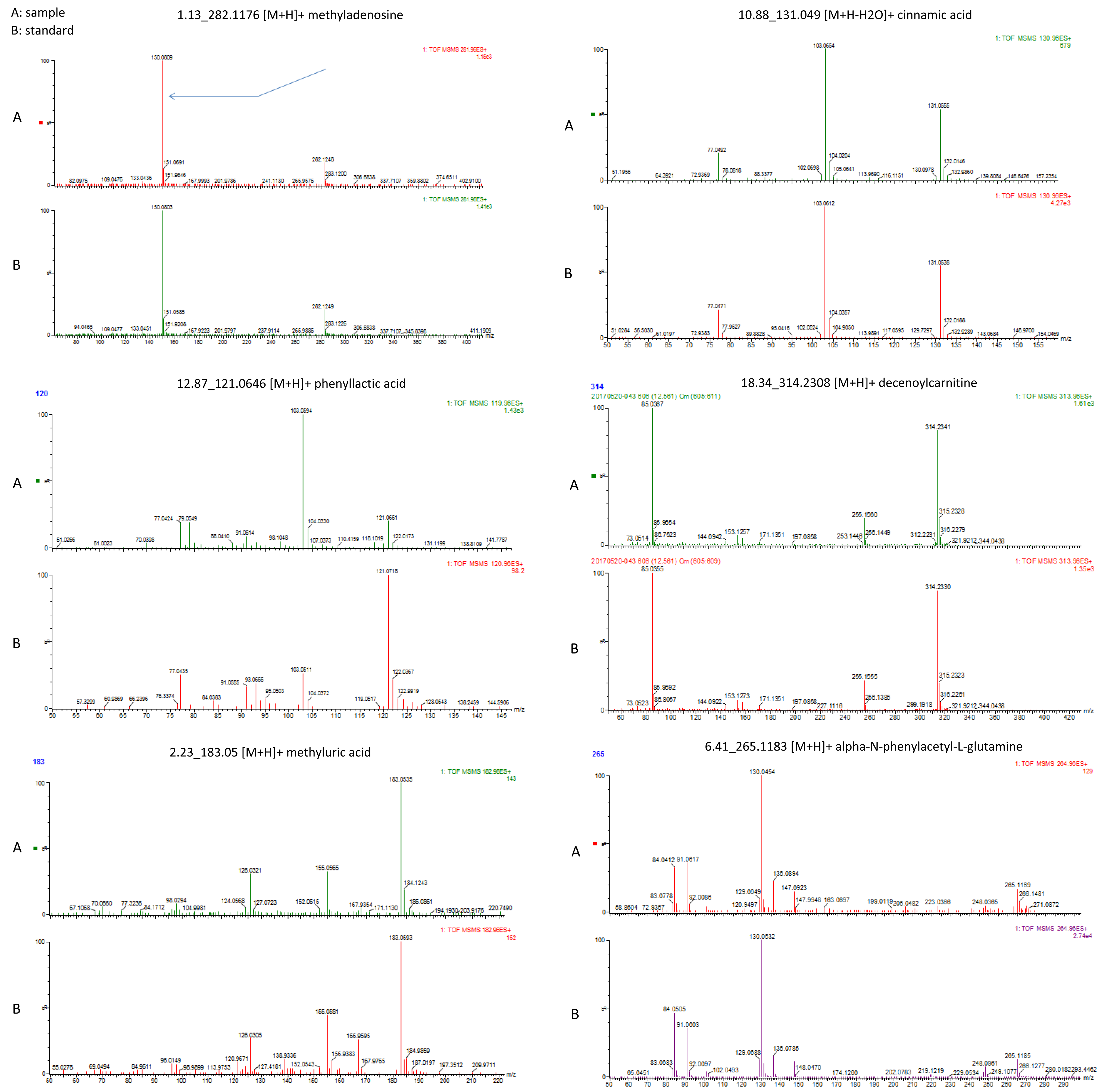

Supplement: FIGURE S3 — MS/MS spectra of six identified metabolites in samples (A) and standards (B). [file Image_3.TIF]

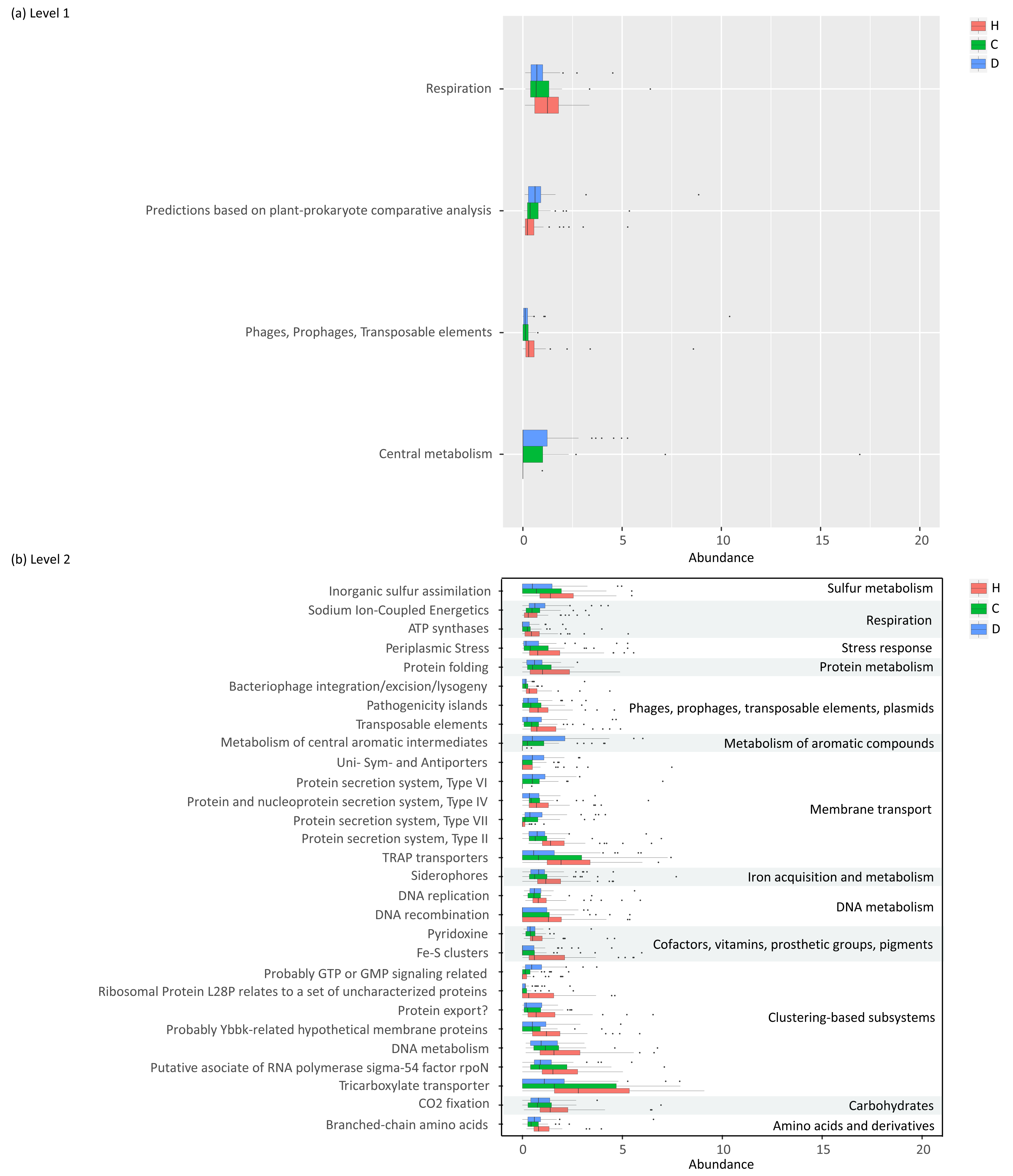

Supplement: FIGURE S4 — Functions varied during liver cirrhosis progression at level 1 (A) and level 2 (B). [file Image_4.TIF]

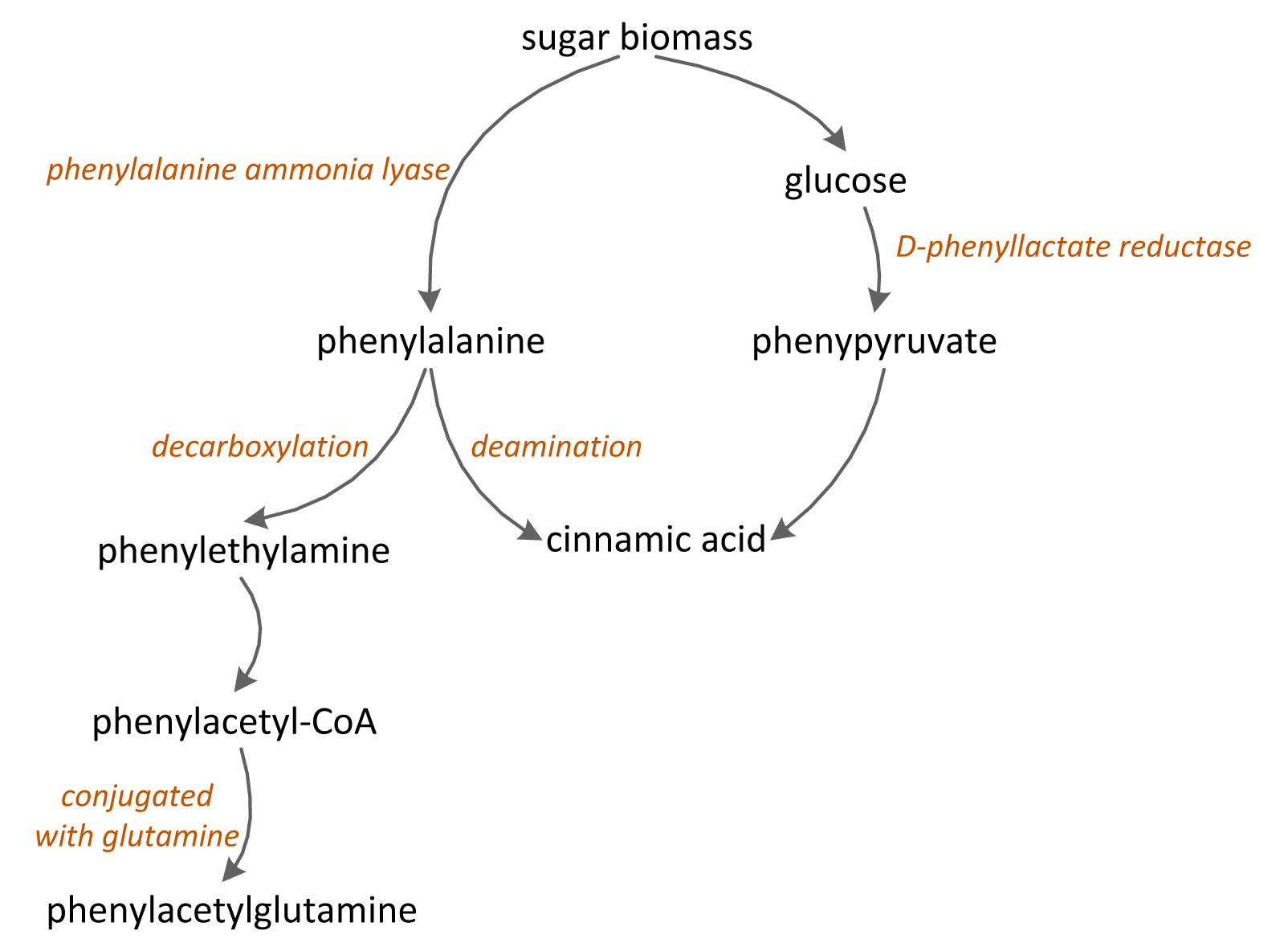

Supplement: FIGURE S5 — Schematic illustration of specific pathways in sugar biomass fermentation. [file Image_5.TIF]
